# Case Report: Confirmed and suspected heparin-induced thrombocytopenia during therapeutic plasma exchange in neuromyelitis optica spectrum disorder: a single-centre case series

**DOI:** 10.3389/fimmu.2026.1884839

**Published:** 2026-09-14

**Authors:** Aleksandra Podlecka-Piętowska, Joanna Rychter, Kinga Sujkowska, Beata Zakrzewska-Pniewska, Monika Nojszewska

**Affiliations:** Department of Neurology, Medical University of Warsaw, Warsaw, Poland

**Keywords:** AQP4 antibodies, heparin-induced thrombocytopenia (HIT), neuromyelitis optica spectrum disorder (NMOSD), PF4 antibodies, plasma exchange (PE), thromboembolism prophylaxis

## Abstract

Neuromyelitis optica spectrum disorder (NMOSD) is a B-cell–mediated autoimmune disease associated with aquaporin-4 (AQP4-IgG) antibodies in which patients may require anticoagulation, including heparin for venous thromboembolism prophylaxis, thereby exposing them to a risk of heparin-induced thrombocytopenia (HIT), an immune-mediated complication caused by antibodies directed against PF4–heparin complexes. Whether anti-AQP4–mediated autoimmunity contributes to susceptibility to anti-PF4/heparin immune responses warrants further investigation. Four cases of suspected or confirmed HIT in patients with NMOSD are presented, together with a review of the current literature. In our NMOSD cohort (n=90), 24 patients underwent therapeutic plasma exchange (TPE), among whom HIT was suspected in four cases (16,7% suspected and 8.3% immunoassay positive). These findings underscore the need for vigilance regarding HIT and thromboembolic complications in NMOSD patients undergoing relapse treatment with TPE. Strategies for the safe continuation of TPE using non-heparin anticoagulation and considerations for outpatient management are also addressed. Awareness of this potential association may facilitate earlier recognition and appropriate management in clinical practice.

## 1 Introduction

Neuromyelitis optica spectrum disorder (NMOSD) is an autoimmune astrocytopathy characterised by demyelinating lesions predominantly affecting the optic nerves, spinal cord, and brainstem. Serum aquaporin-4 (AQP4)-IgG antibodies play a central pathogenic role and are present in most patients (1). The prevalence of NMOSD is estimated to range from approximately 0.5 to 4 per 100,000, with a marked female predominance (female-to-male ratio of about 9:1). Current diagnostic criteria are based on the 2015 consensus by Wingerchuk et al. (2), although updated recommendations have been proposed at the 2025 ECTRIMS Congress (3). As these late-breaking guidelines remain preliminary, the 2015 IPND criteria remain the current diagnostic standard. Immunosuppressive and immunotherapeutic strategies are recommended to prevent relapses that can result in severe disability (4).

Anticoagulation is often required during the course of NMOSD, particularly for venous thromboembolism prophylaxis and during therapeutic plasma exchange (TPE) performed for relapse treatment (5). However, exposure to heparin carries a risk of heparin-induced thrombocytopenia (HIT), an immune-mediated adverse reaction caused by antibodies directed against platelet factor 4–heparin complexes (anti-PF4/heparin Abs) (6). HIT is associated with both venous and arterial thrombosis and can be life-threatening.

This article describes the co-occurrence of two rare conditions, NMOSD and HIT, in the context of the available literature. Between 2019 and 2025, four cases of confirmed or probable HIT were identified among patients with NMOSD treated at the Department of Neurology, Medical University of Warsaw.

## 2 Methods

A retrospective analysis was performed of patients with NMOSD (n=90) treated at our institution between 2019 and 2025. Patients were identified from a prospectively maintained institutional database approved by the Bioethics Committee of the Medical University of Warsaw. Among the 24 patients who underwent TPE for NMOSD relapse treatment, four cases of suspected or confirmed HIT were identified and included in this study. Clinical data, including demographic characteristics, NMOSD therapies, heparin exposure, TPE procedures, platelet count dynamics monitored systematically at regular intervals (with assessments performed on alternate days), HIT diagnostic evaluation, management, and clinical outcomes, were retrospectively collected and analysed.

TPE was performed by membrane filtration using unfractionated heparin (UFH) for circuit anticoagulation (5,000 IU intravenous bolus followed by a continuous infusion of 2,000 IU/h). Albumin (50 mL/kg body weight) was used as the replacement fluid. Five to seven TPE sessions were initially planned on alternate days, with the final number and timing determined by the patient’s clinical condition. Following suspected HIT in Case 4, TPE was switched to centrifugal apheresis using citrate anticoagulation. The timing of TPE procedures is shown in **Figures 1**–**3**.

**Figure 1:**
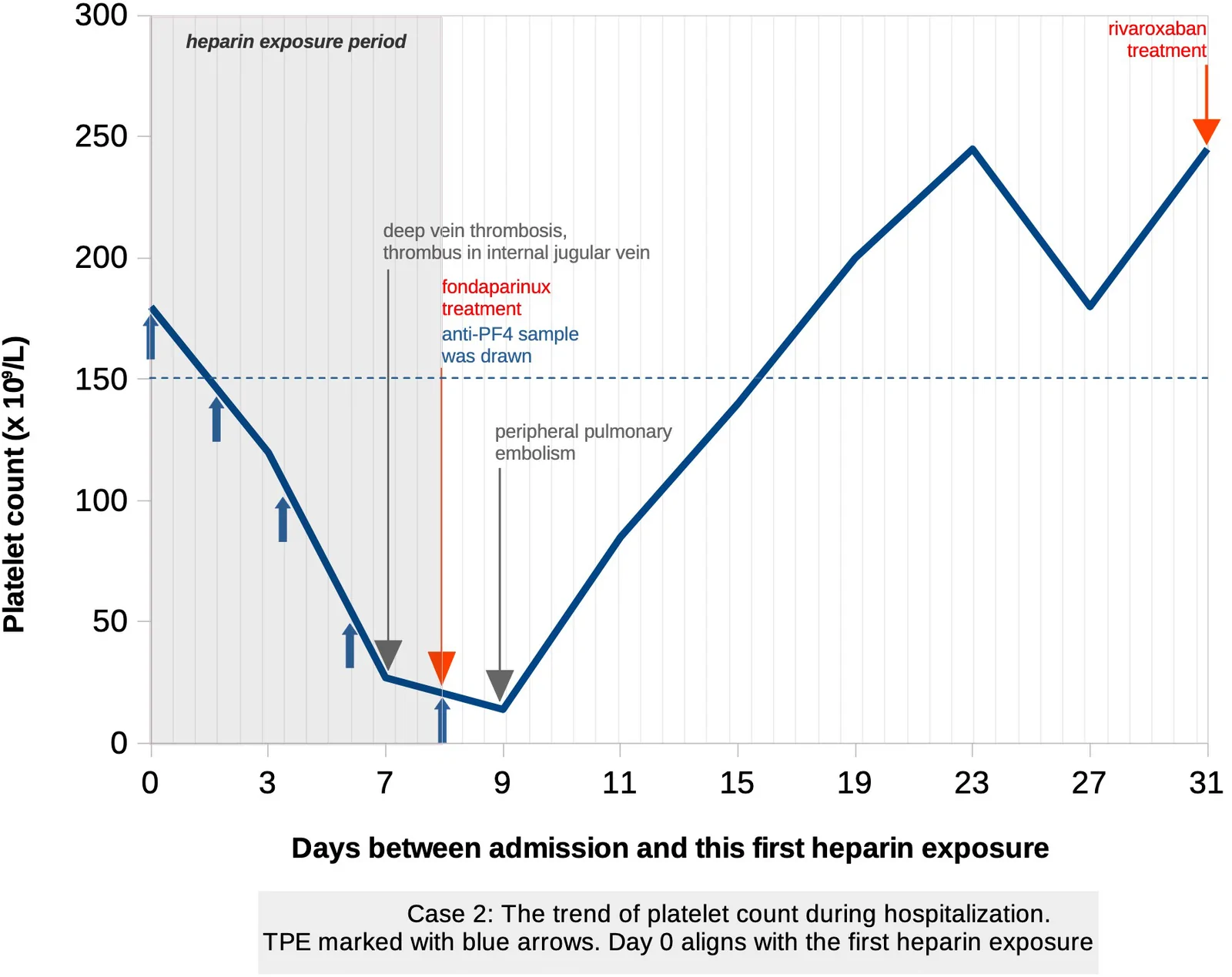
Case 2: the trend of platelet count during hospitalization. TPE marked with blue arrows. Day 0 aligns with the first heparin exposure.

**Figure 2:**
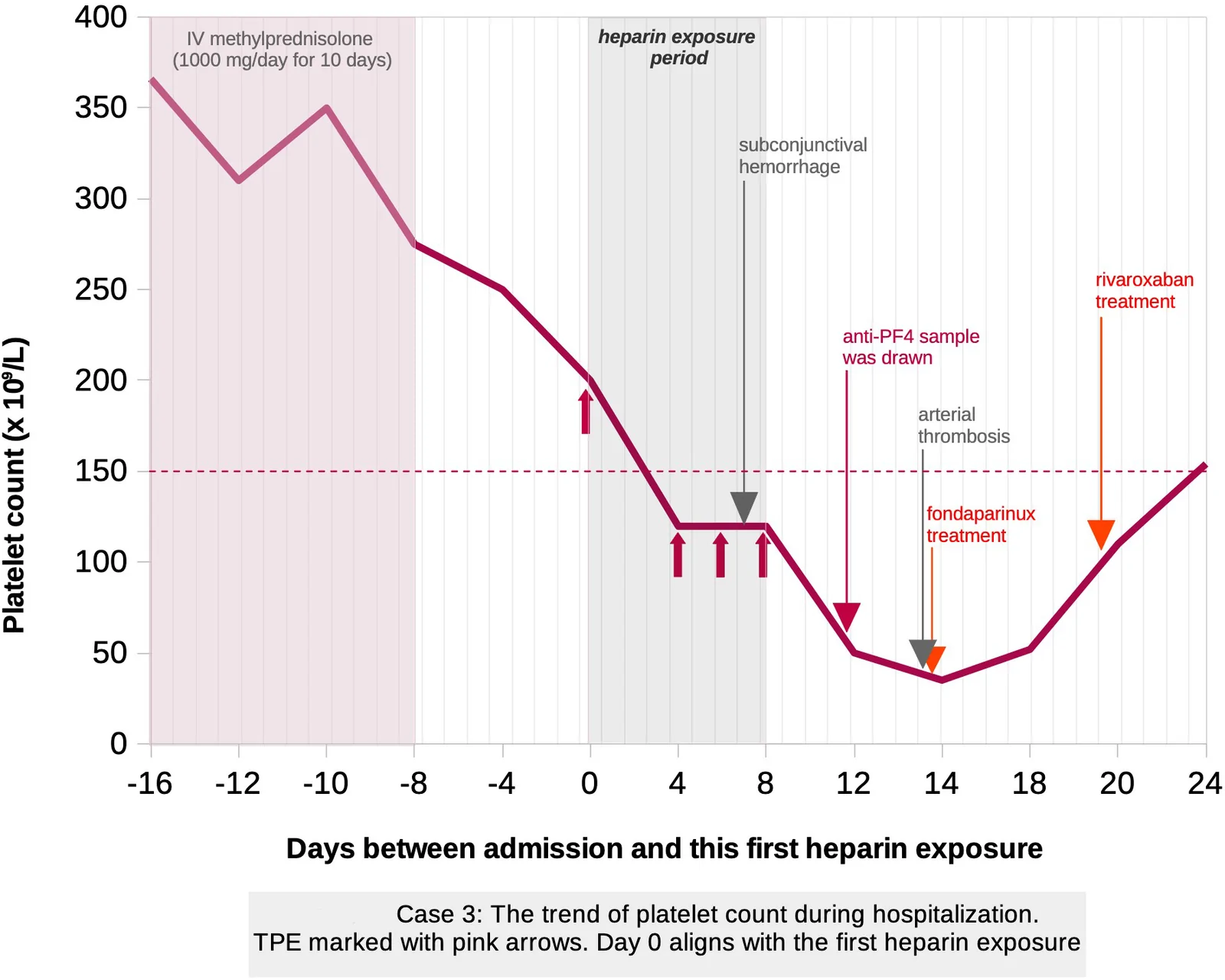
Case 3: the trend of platelet count during hospitalization. TPE marked with pink arrows. Day 0 aligns with the first heparin exposure.

**Figure 3:**
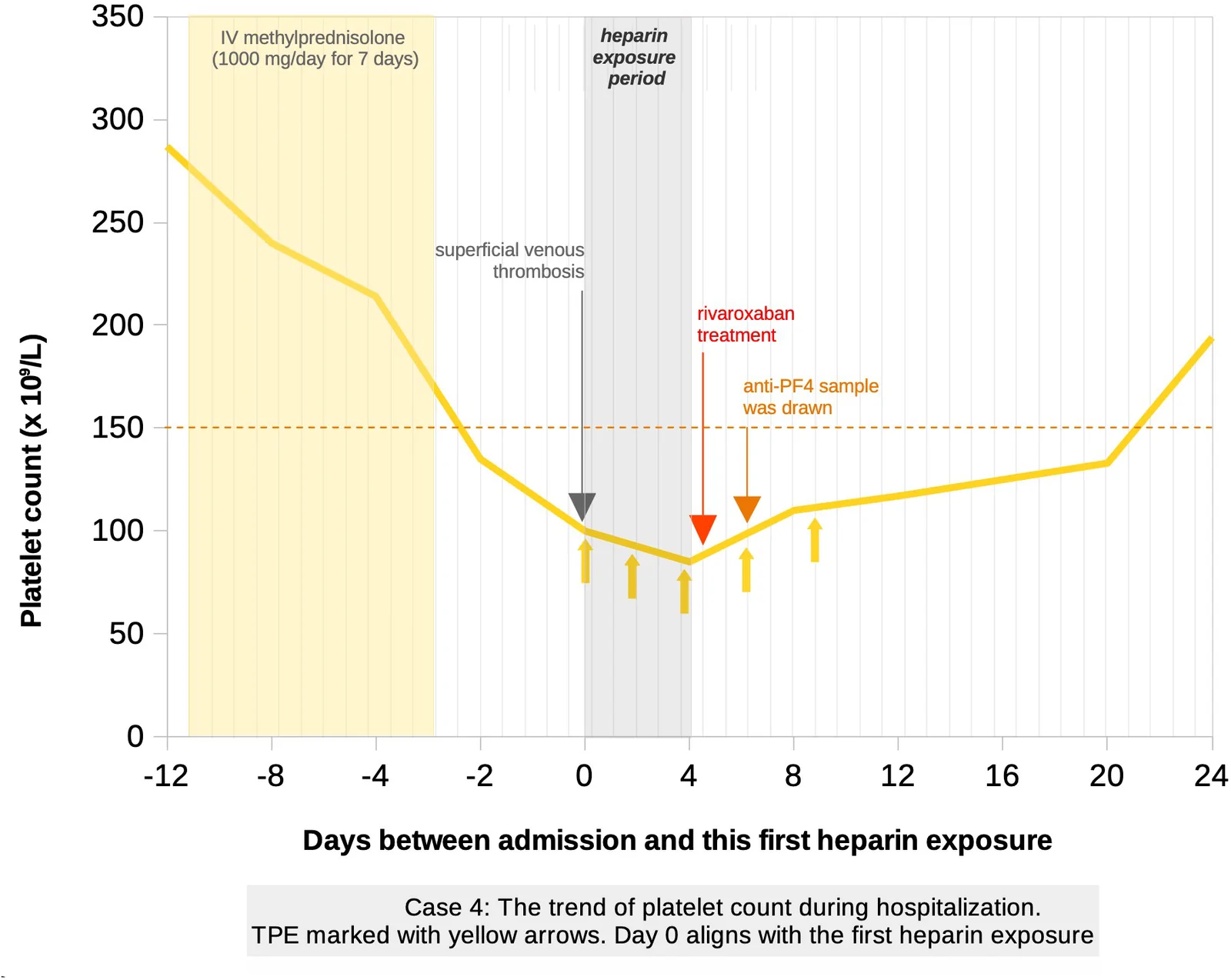
Case 4: The trend of platelet count during hospitalization. TPE marked with yellow arrows. Day 0 aligns with the first heparin exposure.

Patients with suspected HIT underwent haematological consultation, and the pretest probability of HIT was assessed using the 4Ts score. Those with an intermediate or high 4Ts score underwent anti-PF4/heparin Abs testing. Anti-PF4/heparin IgG Abs were measured using a commercially available ELISA (Immucor, USA). According to the manufacturer’s instructions, an optical density (OD) value >0.4 was considered positive. Positive immunoassay results were obtained in Cases 2 and 3.

Written informed consent was obtained from the patient for publication of this case report.

## 3 Case reports

### 3.1 Case 1

A 44-year-old woman with seropositive NMOSD diagnosed in July 2019 was transferred to our unit in August 2019 due to steroid-refractory relapse with spastic paraplegia, sensory impairment, and urinary retention (EDSS 7.0). Clinical deterioration was associated with thoracic spinal cord lesions on magnetic resonance imaging (MRI). Laboratory tests were unremarkable at admission. Due to progressive sphincter dysfunction, seven TPE sessions were initiated on alternate days. During hospitalisation, the platelet count decreased from 389 × 10^9^/L at admission to a nadir of 58 × 10^9^/L, representing an 85% decrease. The 4Ts score was estimated at 5–7, depending on the timing of platelet nadir, which could not be precisely determined; anti-PF4/heparin antibody status was unavailable, and these data are therefore not presented in the Figures. Ultrasound confirmed deep vein thrombosis of the right lower limb. Following suspicion of HIT, heparin was discontinued, TPE was suspended, and fondaparinux (7.5 mg once daily) was initiated. Platelet count recovered to 329 × 10^9^/L by the time of discharge. Due to suspected HIT, the patient was subsequently transitioned to rivaroxaban 20 mg once daily and remains under outpatient care at a vascular diseases clinic. During follow-up, the patient reported gradual clinical improvement, with EDSS decreasing to 6.5 after relapse treatment. Since 2019, the patient has remained on rituximab with sustained clinical remission (current EDSS 5.0) and continues rivaroxaban due to previous lower-limb venous thromboembolism. Anti-PF4/heparin IgG status was not reassessed during follow-up.

### 3.2 Case 2

In July 2021, a 53-year-old woman with seropositive NMOSD diagnosed in 2016 was admitted due to a steroid-refractory relapse characterised by upper limb paresthesias, severe lower limb weakness, and vomiting (EDSS 8.5). MRI revealed new demyelinating lesions in the brain and cervical spinal cord. Five TPE sessions were initiated on alternate days. Admission laboratory tests were unremarkable. From the initiation of TPE, platelet counts progressively decreased from 175 × 10^9^/L at admission, reaching a nadir of 14 × 10^9^/L on day 10 of heparin, representing a 92% decrease from baseline (**Figure 1**). Ultrasound demonstrated asymptomatic deep vein thrombosis in the left lower limb and an asymptomatic thrombus in the right internal jugular vein. Chest computed tomography (CT) revealed an asymptomatic peripheral pulmonary embolism. The 4Ts score was 7, indicating a high probability of HIT. Anti-PF4/heparin Abs testing was performed on day 8 of heparin exposure, with a positive anti-PF4/heparin IgG ELISA result. Anticoagulation was subsequently switched to fondaparinux 7.5 mg once daily. TPE was discontinued after five sessions (EDSS 7.0 after relapse treatment). Following initiation of fondaparinux, platelet counts normalised within 16 days, reaching 247 × 10^9^/L at discharge, without clinical complications. The patient was subsequently transitioned to apixaban 5 mg twice daily and remains under the care of our department; she remains on immunosuppressive treatment with rituximab. The patient showed significant neurological improvement, with independent ambulation and current EDSS 5.0. She reported improvement in both physical and emotional well-being after initiation of appropriate treatment. Due to previous pulmonary embolism and venous thromboembolism, long-term anticoagulation with apixaban 2.5 mg twice daily was continued after completion of the therapeutic phase (5 mg twice daily).

### 3.3 Case 3

A 64-year-old woman with seronegative NMOSD diagnosed in November 2023 was admitted due to a relapse. The initial presentation in July 2023 included acute right lower limb paresis and hypoesthesia below the Th5 level. Spinal MRI revealed longitudinally extensive transverse myelitis (LETM) from Th1 to Th3 with contrast enhancement. Serum anti-AQP4 and myelin oligodendrocyte glycoprotein antibodies (anti-MOG) were negative on fixed cell-based assays (CBA). Cerebrospinal fluid analysis was unremarkable, and infectious workup, including Borrelia testing, was negative. Chest computed tomography (CT) showed no abnormalities. Following intravenous methylprednisolone treatment, the patient achieved gradual neurological improvement, although EDSS remained unchanged (6.5 at admission and discharge). She was discharged with oral methylprednisolone tapering. After rehabilitation, significant functional improvement was observed (EDSS 3.0–3.5).

In November 2023, the patient was admitted with a second relapse presenting with spastic paraparesis, hypoesthesia below T7 including the anogenital region, and urinary dysfunction (EDSS 3.5). Follow-up MRI showed a longitudinally extensive thoracic spinal cord lesion (T1–T4) with contrast enhancement and a few small demyelinating brain lesions. Anti-AQP4 and anti-MOG Abs were reassessed using a fixed CBA and remained negative; however, testing was performed during prolonged corticosteroid therapy, which may have affected sensitivity. Due to insufficient response to intravenous methylprednisolone (1000 mg/day for 10 days), five TPE sessions were initiated on alternate days. A total of 7 days after initiation of heparin anticoagulation for TPE, subconjunctival haemorrhage was observed. Platelet counts decreased from 366 × 10^9^/L at admission and 200 × 10^9^/L before heparin exposure to a nadir of 34 × 10^9^/L on day 14 of heparin (83% decrease; **Figure 2**), leading to discontinuation of TPE. HIT was suspected; the 4Ts score was 7, indicating high probability. CT angiography revealed acute left popliteal artery thrombosis. Due to severe thrombocytopenia, surgical intervention was not performed. Anticoagulation was switched to fondaparinux (5 mg once daily), and aspirin (75 mg once daily) was added after multidisciplinary haematology and vascular surgery consultation. Anti-PF4/heparin IgG ELISA performed on day 11 of heparin exposure was positive. Platelet counts recovered to 166 × 10^9^/L at discharge, and anticoagulation was continued with rivaroxaban (20 mg once daily). Neurological status improved, with EDSS remaining stable at 3.5 at discharge. Based on the clinical, radiological, and laboratory findings (negative OCB type 2, serum anti-AQP4, anti-MOG, ANA, and infectious workup), a diagnosis of seronegative NMOSD was established. Neuroimaging findings fulfilled the 2015 IPND criteria, demonstrating LETM during the first relapse (Th1–Th3) and a subsequent new LETM lesion extending from Th1 to Th4, followed by spinal cord atrophy, consistent with NMOSD. Due to the active and fulminant disease course, rituximab therapy was initiated. The patient remains under haematological and vascular surgery follow-up, and CT angiography showed progressive resolution of thromboembolic lesions. Rivaroxaban was continued until March 2025. The patient remained clinically stable, with gradual improvement in daily functioning (current EDSS 2.0).

### 3.4 Case 4

A 37-year-old man with seronegative NMOSD was admitted in January 2025 with right optic neuritis (EDSS 3.5). His previous relapses (2016–2018) included motor and sensory symptoms, with MRI showing cervical spinal cord lesions (multiple, confluent demyelinating lesions C2–C5 with heterogeneous contrast enhancement and mild oedema) and limited brain lesions. Cerebrospinal fluid analysis performed in 2016 demonstrated the presence of type 2 oligoclonal bands. Serum anti-AQP4 and anti-MOG Abs tested negative using fixed CBA. Differential diagnosis included multiple sclerosis (MS) and NMOSD. He had not received disease-modifying therapy due to personal preference.

At admission in 2025, orbital MRI demonstrated longitudinally extensive right optic nerve enhancement, while brain MRI showed few T2-hyperintense lesions with mild corpus callosum thinning. Spinal MRI revealed confluent cervical demyelinating lesions (C2–C5) and marked atrophy. Visual evoked potentials confirmed right optic nerve dysfunction (absent response). Due to poor response to intravenous methylprednisolone (1000 mg/day for 7 days), five TPE sessions were planned on alternate days. On the day of the first TPE session, superficial venous thrombosis of the right arm was diagnosed. From the day of admission, platelet counts progressively decreased from 287 × 10^9^/L and 135 × 10^9^/L before heparin exposure to a nadir of 85 × 10^9^/L on day 6 after heparin initiation (37% decrease; **Figure 3**). Haematological assessment resulted in a 4Ts score of 5, indicating an intermediate probability of HIT. Based on this assessment, rivaroxaban was initiated, and TPE was continued using citrate anticoagulation. Anti-PF4/heparin IgG ELISA performed on day 6 of heparin exposure was negative (functional assays were unavailable at that time). Platelet counts recovered after discontinuation of heparin exposure during TPE, reaching 232 × 10^9^/L at discharge. Based on the clinical presentation, haematological consultation supported the diagnosis of probable HIT, and future avoidance of heparin products was recommended.

During hospitalisation, anti-AQP4 and anti-MOG Abs were reassessed using fixed CBA (live CBA was unavailable in Poland) and remained negative at baseline and after 3 months of follow-up. Testing was performed during prolonged corticosteroid therapy, which may have affected assay sensitivity. The patient’s first neurological symptoms occurred in 2016, and during follow-up until 2025, MRI examinations did not demonstrate features suggestive of MS progression, including progression independent of relapse activity (PIRA) or brain atrophy, which are typical for MS. In contrast, marked spinal cord atrophy was observed following longitudinally extensive spinal cord lesions, which is consistent with the 2015 IPND criteria supporting an NMOSD diagnosis. Regarding MS-specific imaging markers, the available brain MRI data did not reveal central vein sign (CVS) or paramagnetic rim lesions (PRLs), which are increasingly recognised as supportive biomarkers of MS according to the 2024 revision of the McDonald criteria. Clinically, the patient presented with severe, treatment-refractory optic neuritis characterised by longitudinally extensive optic nerve involvement, a pattern typical of NMOSD. Neuroimaging did not reveal features suggestive of MOG-associated disease, such as optic nerve perineuritis. Additionally, visual evoked potentials demonstrated absent responses from the affected eye, supporting severe optic nerve injury. Cerebrospinal fluid analysis revealed type 2 oligoclonal bands, which, although more commonly associated with multiple sclerosis, may also be present in a subset of NMOSD patients (1). Based on the clinical, radiological, and laboratory findings, seronegative NMOSD was diagnosed, and rituximab therapy was initiated due to active disease. Follow-up care in haematology and neurology outpatient clinics was arranged; anti-PF4/heparin IgG serostatus was not reassessed. Following initiation of rituximab therapy, the patient achieved clinical stabilisation of NMOSD, with EDSS remaining at 3.5. Rivaroxaban was discontinued after 1 month of treatment based on recommendations from a haematologist and vascular surgeon.

## 4 Discussion

HIT is a rare condition with an incidence ranging from 0.1% to 7% among hospitalised patients (7). The coexistence of NMOSD and HIT, both rare conditions, is therefore noteworthy. In our unit, 90 patients with NMOSD were followed; among 24 NMOSD patients undergoing TPE (**Table 1**), HIT was suspected in 4 cases (16,7%) and immunoassay positive in 2 cases (8,3%). The most recent and largest directly relevant series by Lv et al. (2025) (8) reported that among 158 patients with neurological autoimmune diseases undergoing TPE, 139 developed a significant decrease in platelet count; however, only 23 patients with a continuous platelet decline and 4Ts scores ≥ 4 underwent PF4 antibody testing, and of these only 4 tested positive (2.5%). Clinical reports have suggested a possible association between inflammatory neurological diseases, such as NMOSD or MS, and an increased risk of HIT; However, the current evidence remains limited, precluding definitive conclusions (7, 9).

**Table 1:** Patients with NMOSD treated with TPE (n=24).

Variable	Without HIT suspicion (n=20)	HIT suspected or confirmed (n=4)
Mean age (years)	51.3	49.5
Mean disease duration (years)	8.9	6
Mean baseline EDSS	5.5	5.5
Positive AQP4-IgG serostatus (n)	16	2
Core clinical manifestation (n)		
• optic neuritis	18	1
• myelitis	16	3
• area postrema syndrome	9	0

Although most patients with NMOSD test positive for AQP4-IgG or MOG-IgG, there exists a subset of double-seronegative patients who share similar clinical and radiological features (10). The pathophysiology of NMOSD is likely driven by a heterogeneous inflammatory response, with the primary mechanism involving B-cell–mediated injury with AQP4-IgG binding to astrocytes, complement system activation, subsequent antibody-dependent cell-mediated cytotoxicity (ADCC), and inflammation. Given the chronic inflammation associated with this condition, it may trigger procoagulant responses (9, 11).

In standard relapse management, escalation to TPE is recommended in patients with incomplete recovery following intravenous glucocorticosteroid therapy, which remains the first-line treatment. Given the high risk of permanent disability, aggressive treatment of acute attacks followed by maintenance immunosuppressive therapy is recommended (13). In this context, serological status assessment may be false-negative if performed during immunosuppression or shortly after relapse treatment. Of note, a recently recommended diagnostic method, the live CBA (not commercially available in Poland), demonstrates the highest sensitivity and specificity for the serological detection of NMOSD and may be particularly useful in patients with low AQP4-IgG titres or borderline results (3). In our two seronegative cases, diagnostic tests were performed after the initiation of treatment; therefore, the true serological status of these patients remains unknown.

In the pathophysiology of HIT, IgG antibodies develop against complexes of PF4 and heparin. It is important to note that even heparin “flushes,” i.e., small doses of heparin used to maintain catheter patency, may be sufficient to trigger HIT (14). According to Arepally et al. (6), anti-PF4/heparin Abs assemble into ultralarge antigenic complexes that trigger prothrombotic responses with platelet activation via FcγRIIa and subsequent propagation to endothelial cells, monocytes, and coagulation proteins. Nevertheless, there is no evidence of direct endothelial activation; however, substantial data support PF4–heparin immune complex–mediated bystander injury as a key pathogenic mechanism (6, 15). In severe cases, thrombocytopenia may develop within 24 hours of heparin exposure, likely reflecting the presence of pre-existing circulating HIT antibodies due to prior exposure to heparin within the preceding 100 days. Moreover, polymorphic variation in the extracellular domain of the FcγRIIa receptor and its functional regulation have been identified as potential genetic factors influencing susceptibility to thrombosis in HIT (6). Thrombocytopenia is the hallmark of HIT (16); however, the mechanisms underlying its development remain incompletely understood in the current literature.

The risk of HIT depends on several variables, including the type of heparin (higher for UFH vs low-molecular-weight heparin [LMWH]), the duration of heparin exposure, and the patient population (higher incidence for age over 40 and in males), with a higher risk observed in surgical patients (12, 17). These determinants influence the likelihood of seroconversion, which typically occurs 5–10 days after heparin exposure. In addition, prothrombotic conditions such as tissue injury and inflammation may elevate the risk of this life-threatening complication (16). Moreover, venous thromboembolism (VTE) in patients with NMOSD has been reported predominantly in those with transverse myelitis (18), in whom immobility, hospitalisation, corticosteroid therapy, and repeated venous access are strong, independent risk factors for VTE. In our patients, EDSS scores, also reflecting the ability to ambulate independently, are presented in **Table 2**.

**Table 2:** Patient characteristics.

Variable	Case 1	Case 2	Case 3	Case 4
Age	44	53	64	37
Sex	Female	Female	Female	Male
NMOSD: AQP4-IgG (assay, timing)	positive (fixed CBA; 2019)	positive (fixed CBA; 2021)	negative (fixed CBA, 09/2023, 11/2023, 2025)	negative (fixed CBA, 2016, 01/2025, 03/2025)
NMOSD: MOG-IgG (assay, timing)	N/A	negative (fixed CBA; 2021)	negative (fixed CBA, 09/2023, 11/2023, 2025)	negative (fixed CBA, 2016, 01/2025, 03/2025)
NMOSD 2015 IPND criteria	• severe relapse with spastic paraplegia • MRI: LETM, confluent lesion (Th2-Th6), oedema, contrast enhancement • anti-AQP4 Abs detected • differential diagnostic workup: negative	• area postrema syndrome and spastic tetraparesis • MRI: LETM, confluent lesion from brainstem to C6, oedema, contrast enhancement • anti-AQP4 Abs detected • differential diagnostic workup: negative	• relapse: right lower limb paresis, truncal hypoaestesia; MRI: LETM (T1–T3), confluent contrast-enhancing lesion • relapse: spastic paraparesis, urinary dysfunction; MRI: LETM (T1–T4), confluent contrast-enhancing lesion; follow-up: spinal cord atrophy • differential diagnostic workup: negative	• relapse: left upper limb paresis; MRI: predominant spinal cord lesions, oedema, marked atrophy (C2-C5) • relapse: severe treatment-refractory optic neuritis; MRI: longitudinally extensive optic nerve enhancement • differential diagnostic workup: negative
EDSS (relapse)	7.0	8.5	3.5	3.5
EDSS (discharge)	6.5	7.0	3.5	3.5
EDSS (follow-up)	5.0	5.0	2.0	3.5
Heparin exposure in prior 100 days	No	No	No	No
TPE modality	membrane filtration	membrane filtration	membrane filtration	membrane filtration/centrifugal apheresis, citrate anticoagulation
TPE replacement fluid	albumin	albumin	albumin	albumin
TPE sessions (n)	N/A	5	4	5
Heparin (type, dose)	TPE: UFH 5,000 IU IV bolus, infusion 2,000 IU/h			
PLT at admission (10^9^/L)	389	175	366	287
PLT pre-heparin baseline (10^9^/L)	N/A	175	200	135
PLT at nadir (10^9^/L), day of heparin	58 (day of nadir N/A)	14 (10 day)	34 (14 day)	85 (6 day)
% fall from admission	85%	92%	91%	70%
% fall from pre-heparin	N/A	92%	83%	37%
4Ts (thrombocytopenia/timing/thrombosis/other)	5-7 (2+N/A+2 + 1)	7 (1 + 2 + 2+2)	7 (2 + 1 + 2+2)	5 (1 + 2 + 0+2)
Thrombosis (site, imaging)	USG: deep vein thrombosis, right lower limb	• USG: deep vein thrombosis, left lower limb; right internal jugular vein; • CT: peripheral pulmonary embolism	CT: arterial thrombosis, left popliteal artery	superficial venous thrombosis, right arm
Thrombosis (day of heparin)	N/A	day 7 and 9 of heparin	day 13 of heparin	day 1 of heparin
Anti-PF4/H IgG ELISA result (positive OD value > 0.4)	N/A	positive, drawn at day 8 of heparin	positive, drawn at day 11 of heparin	negative, drawn at day 6 of heparin
Functional assay	N/A	N/A	N/A	N/A
Alternative anticoagulant	fondaparinux 7.5 mg/rivaroxaban 20 mg once daily	fondaparinux 7.5 mg/apixaban 5 mg twice daily	fondaparinux 5 mg, ASA 75 mg/rivaroxaban 20 mg once daily	rivaroxaban 20 mg once daily
Weight (kgs)	104	83	50	100
eGFR (ml/min/1,73m2)	103	120	73	80
Day platelets > 150 × 10^9^/L	N/A	day 15 of heparin	day 21 of heparin	day 24 of heparin
Duration of follow-up (from HIT suspicion)	7 years	5 years	3 years	1 year

A key differential diagnostic consideration is distinguishing HIT from the transient platelet decline associated with TPE. This expected reduction is usually predictable, non-immune mediated, and followed by spontaneous platelet recovery within days (8). In the study by Lv et al. (2025) (8), patients with autoimmune neurological diseases undergoing TPE experienced a mean platelet count decline of 37%; nevertheless, confirmed type II HIT was identified in only 4 of 158 patients (2.5%), while most patients showed spontaneous platelet recovery within 4 days. Therefore, a falling platelet count during TPE is the rule, not in itself evidence of HIT, and warrants attention in differential diagnosis. Case 4 illustrates the diagnostic challenge of distinguishing HIT-related thrombocytopenia from the expected platelet decline associated with TPE. Platelet counts decreased progressively from the day of admission, reaching a nadir on day 4 after heparin exposure. Following discontinuation of heparin, conversion to citrate-based centrifugal apheresis, and initiation of rivaroxaban, platelet recovery to >150 × 10^9^/L occurred only on day 21 after heparin exposure.

None of the described patients had undergone recent surgery, blood transfusion, or heparin exposure within the preceding 100 days, making pre-existing anti-PF4/heparin Abs unlikely. Sepsis and drug-induced thrombocytopenia were excluded (19). Following standard haematological algorithms, thrombotic thrombocytopenic purpura (TTP) was considered; ADAMTS13 activity and antibody testing were performed, along with assessment of haemolysis markers (bilirubin, lactate dehydrogenase, and haptoglobin), Coombs tests, and abdominal ultrasound (20). In Case 3, the diagnostic work-up was extended to bone marrow examination and peripheral blood immunophenotyping, with no pathological findings. Additional antiphospholipid syndrome (APS) testing, including anti-cardiolipin antibodies, anti-β2-glycoprotein-I antibodies, and antinuclear antibodies (ANA), was performed only in Case 3 and was negative. The lack of systematic APS evaluation in all patients represents a limitation of this study.

Clinical algorithms for HIT include the 4Ts score, which is used to estimate the probability of HIT by considering the degree of platelet count reduction, the timing of symptom onset, the presence of new thrombosis, and the exclusion of other causes of thrombocytopenia. Further diagnostic testing is recommended for intermediate and high probability of HIT (15, 16). Statistically, patients develop thrombocytopenia between days 4 and 14 after the initiation of heparin therapy (6). Clinical manifestations in one-third to a half of all patients include thromboses in large vessels and microvascular thrombosis, with careful evaluation required for rare cases of localised thrombosis, such as skin necrosis. Notably, Warkentin et al. reported that almost half of HIT cases may be recognised only after a new thrombotic event (21).

Clinical diagnosis of HIT should be confirmed with laboratory assays. Immunoassays (e.g., ELISA) detect circulating anti-PF4/heparin Abs regardless of their capacity to activate platelets and are characterised by high sensitivity, but low specificity (16). In patients with systemic diseases (e.g., systemic lupus erythematosus), false-positive results may occur (9). As mentioned above, in a B-cell–mediated autoimmune population, the apparent excess of HIT may partly reflect false-positive immunoassay results, which represents a limitation of this study. Functional assays, which have higher specificity than immunoassays (14), detect heparin-dependent platelet-activating antibodies in patient plasma or serum, with the serotonin release assay (SRA) being the most used. Another relevant test is the heparin-induced platelet activation (HIPA) assay, both of which, SRA and HIPA, offer higher specificity than PF4-dependent immunoassays such as enzyme immunoassays (EIAs) (12, 14, 16). However, functional assays are not widely available, including in Poland. In our case series, HIT diagnosis was based on ELISA testing. A limitation of this study is the lack of exact anti-PF4/heparin ELISA optical density (OD) values, as only qualitative results (positive/negative) were documented in the medical records.

For non-surgical patients, immediate discontinuation of all heparin products is required upon suspicion of HIT. Acute-phase anticoagulation options include direct thrombin inhibitors (DTIs, e.g., argatroban), direct oral anticoagulants (DOACs), and fondaparinux. Vitamin K antagonists (VKAs) are contraindicated during the acute phase of HIT until platelet recovery to ≥ 150 × 10^9^/L, due to the risk of venous limb gangrene and skin necrosis (12). All aforementioned therapeutic options lack interaction with heparin–PF4 antibodies (15). Treatment with fondaparinux or DTIs should be continued until platelet count recovery (12). In the treatment of our patients, we used fondaparinux due to local availability, with fondaparinux dosing dependent on GFR and the patient’s body weight. Nevertheless, in many clinical settings, argatroban, danaparoid, and bivalirudin remain guideline-supported first-line parenteral options. Subsequent transition to a DOAC appears to be a safe approach (22, 23), with rivaroxaban being the first off-label agent introduced and the greatest support from clinical experience (14). In patients with VTE, DOAC therapy typically lasts 3 to 6 months, depending on the presence of thrombosis on follow-up imaging (24). Compared with VKAs, DOACs do not require routine laboratory monitoring, have a rapid onset of action, and are easy to administer (15, 16).

For secondary prevention, it should be noted that TPE protocols often involve heparin flushes to maintain venous access; therefore, all heparin-containing procedures should be avoided in patients with a history of HIT. In Case 4, TPE sessions were performed using citrate anticoagulation instead of UFH, which may be considered an alternative therapeutic approach (25). Therefore, there is no need to discontinue TPE in HIT when a heparin-free (citrate-based) circuit is used; TPE continuation is currently recommended rather than cessation (16, 25). It is worth noting that TPE itself is a recognised treatment modality in HIT as an initial immunomodulatory strategy, highlighting its dual role as both a potential trigger and a therapeutic tool, e.g., to facilitate heparin exposure during cardiopulmonary bypass, alongside intravenous immunoglobulins (IVIG), which are most commonly used for the treatment of refractory HIT (26).

## 5 Summary

In our NMOSD cohort (n=90), HIT was suspected in 4 of 24 patients undergoing TPE (16,7%), including 2 immunoassay-positive cases (8.3%). Although causality cannot be inferred, these observations highlight the need for vigilance regarding HIT and associated thromboembolic complications in patients with severe NMOSD relapses requiring TPE. If HIT is suspected, TPE may be safely continued using a heparin-free (citrate-based) circuit. Whether or not anti-AQP4–mediated autoimmunity contributes to susceptibility to anti-PF4/heparin Abs immune responses warrants further investigation.

## Data Availability

The datasets presented in this article are not readily available because of ethical and privacy restrictions. Requests to access the datasets should be directed to the corresponding author.
